# Transradial approach for the endovascular treatment of type I endoleak after aortic aneurysm repair: a case report

**DOI:** 10.1186/1471-2482-13-S2-S47

**Published:** 2013-10-08

**Authors:** Gabriele Giacomo Schiattarella, Fabio Magliulo, Flora Ilaria Laurino, Roberta Bottino, Antonio Giulio Bruno, Michele De Paulis, Antonio Sorropago, Cinzia Perrino, Bruno Amato, Dario Leosco, Bruno Trimarco, Giovanni Esposito

**Affiliations:** 1 Department of Advanced Biomedical Sciences, Federico II University, via Pansini 5, 80131 Naples; Italy; 2 Department of Clinical Medicine and Surgery, Federico II University, via Pansini 5, 80131 Naples, Italy; 3 Department of Translational Medical Sciences, Federico II University, via Pansini 5, 80131 Naples, Itlay

## Abstract

**Background:**

Endovascular repair of aortic aneurysms (EVAR) is obtained through the positioning of an aortic stent-graft, which excludes the aneurysmatic dilation. Type I endoleak is the most common complication, and it is caused by an incompetent proximal or distal attachment site, causing the separation between the stent-graft and the native arterial wall, and in turn creating direct communication between the aneurysm sac and the systemic arterial circulation. Endoleak occurrence is associated with high intrasac pressures, and requires a quick repair to prevent abdominal aortic aneurysm rupture.

**Case presentation:**

We report the first case of a 80-year-old man undergoing percutaneous closure of a peri-graft endoleak (type I) by transcatheter embolization through radial arterial access.

**Conclusion:**

The transradial approach has been shown to be a safe and effective alternative to the traditional transfemoral approach. A decrease in vascular complications and improved patient comfort are the primary benefits of this technique in patients with previous EVAR.

## Background

Peripheral arterial disease (PAD) is one of the most frequent manifestations of atherosclerosis and it is associated to a high incidence of major cardiovascular events [1-5]; it might involve the abdominal aorta, the epi-aortic trunks and the limb arteries [6-8]. Aortic abdominal aneurysms (AAA) are frequent expressions of PAD [9] and represent a frequent cause of mortality and morbidity, particularly in men older than 60 years. AAA particularly affect the iuxtarenal portion of the abdominal aorta and are defined as an increase of the antero-posterior diameter of the aorta of more than 3 cm [10]. Risk factors for developing an AAA are the same of atherosclerosis, in particular the relative risk seems to significantly increase with the number of daily smoked cigarettes [11]. AAA are associated with a high risk of rupture, which is frequently fatal [2,12], so it is necessary to perform preventive measures to avoid such eventuality. Most recent guidelines suggest a threshold of 5.5 cm as anteroposterior aortic diameter to decide for clinical surveillance (when inferior) or for operative measures (when superior), even though AAA with a diameter of 5 cm may be considered for intervention in particular cases [2,13].

The classical intervention for treating an AAA is the surgical excission of the dilated sac and the use of a prosthesis, generally made in Goretex, to restore the vascular continuity; however, despite the improving in surgical and anesthetic techniques, this complex procedure still carries an high peri-operative risk [14,15]. Recently, endovascular aortic repair (EVAR) has been proposed as an attractive alternative for selected patients since 1991, when Parodi et al performed the first endovascular positioning of an aortic endoprosthesis excluding the aneurysmatic dilation [16]. Although EVAR short and mid-term results indicate that this procedure is effective and might be even safer than classical open repair, given to the lack of long term follow up data, it is still considered a second choice compared to classic surgery, rather than an equal alternative [2]. Undoubtedly, EVAR downsides include anatomic limitations making this procedure unsuitable in some cases (renal arteries interested into aneurysm sac; short, angulated AAA etc), thrombotic graft occlusions and stent migrations that might cause a re-enlargement of the previously excluded aneurysm [12]. The most frequent complications of EVAR are intrasac endoleaks, which may be defined as the presence of a continuous blood flow into the excluded sac [17].

Currently, the most commonly accepted classification for endoleaks encompasses five types, on the basis of the origin of the anomalous flow (Table 1). Type I endoleaks occur at either a proximal (type IA) or distal (type IB) incompetent attachment site, and allow a persistent communication between the pressure-filled aortic lumen and the aneurysm sac or excluded portion of the aortic lumen, producing high intrasac pressures that can lead to rupture. Type II endoleaks are the result of retrograde flow from branch vessels (for example, lumbar arteries and the inferior mesenteric artery). Type III endoleaks are due to a junctional leak between two modular segments of stent-grafts or due to a graft fabric disruption. As type I endoleaks, they are associated with measureable increases in aneurysm sac size and require urgent management. Type IV endoleaks are the result of high graft porosity and diffuse microleakage through its interstices, usually within 30 days of implantation, and are rare compared with the frequency of other endoleaks. Finally, the terms "type V endoleaks" and "endotension" have been coined to those circumstances in which the excluded sac continues to enlarge despite the absence of any visible endoleaks on contrast-enhanced computed tomographic scans.

**Table 1:** Endoleaks classification

Type I: Incompetent attachment site leaks	
IA	Proximal end of endograft

IB	Distal end of endograft

IC	Iliac occluder (plug)

**Type II: Branch vessel leaks without attachment site connection**	

IIA	From only 1 patent branch

IIB	From 2 or more patent branches

**Type III: Graft defect**	

IIIA	Junctional leak or modular disconnect

IIIB	Fabric disruption (midgraft hole)

**Type IV Graft wall (fabric) porosity (<30 d after graft placement)**	

**Type V Aneurysm sac enlargement with no detectable endoleak**	

The management of post-operative endoleaks depend on the specific type, and in most cases only type I/III require a rapid repair to prevent aneurysm rupture [12,17]. Standard endovascular treatment options for type IA endoleaks include the insertion of an aortic cuff to extend endograft coverage more proximally, or the placement of a large-caliber balloon-expandable stent (e.g. Palmaz or Sinus) inside the proximal endograft to promote the seal. Standard therapy for type IB endoleaks involves distal extension of endograft coverage. If an endoleak persists despite these measures, definitive therapy may require conventional open surgery, combining visceral artery bypass with stent-graft extension or the use of chimney or periscope grafts to extend proximal and distal landing zones.

Patients who are not eligible for these more complex procedures, because of severe co-morbidities or adverse anatomical factors, may be treated by trans-catheter embolization of the endoleak itself. There is limited published experience of type I endoleak embolization and previous reports have involved coils and n-butyl 2-cyanoacrylate (n-BCA). Onyx (ev3, Irvine, CA, USA) is a relatively novel non-adhesive liquid embolic agent, which is most commonly used to treat intracranial arteriovenous malformations. It is comprised of ethylene vinyl alcohol dissolved in dimethyl sulphoxide (DMSO), an organosulfur compound frequently employed into molecular biology studies [18,19], and suspended micronized tantalum powder to provide contrast for visualization under fluoroscopy [20].

Treatment of EVAR endoleaks is generally performed through femoral arterial access; however, radial access are generally associated to lower costs, fewer vascular complications and earlier ambulation. We describe a first case of a patient undergoing percutaneous closure of a type I peri-graft endoleak by embolization release controlled spirals and liquid embolization Onyx through radial arterial access.

## Case presentation

An 80-year-old man, affected by coronary artery disease, chronic obstructive pulmonary disease, arterial hypertension and dyslipidemia, former heavy smoker, was admitted to our Department on May 5, 2011. Previous Doppler ultrasound exams had shown the presence of an aneurysmatic dilatation of the infra-renal portion of the aorta, which had been followed-up until reaching a maximum diameter of almost 5.2 cm (January 2011). Thus, the patient underwent computed tomography (CT) angiographic imaging, which demonstrated aneurysm sac shrinkage of about 5 × 5.5 cm, with a longitudinal extension of about 8 cm and showed also signs of mural thrombotic apposition of about 3 cm of maximum assial thickness.

Physical examination evidenced a tender and pulsatile abdominal mass, accompanied by the auscultatory finding of a systolic murmur in the paraumbilical region. During the hospitalization, aortic ecotomography was repeated and revealed maximum diameters of the aneurysmatic formation of about 6 × 5.8 cm, confirming the presence of a large, eccentric intra-sac thrombus. The patient also underwent coronary angiography, which showed significant stenosis of the left anterior descending coronary artery and of the circumflex coronary artery, which were judged not amenable of percutaneous treatment. Given the size of the aneurysm sac (and, in particular, the progressive and quick enlargement), the presence of intramural thrombotic burden causing a not trivial potential risk of a severe peripheral embolism and the severe coronary artery disease, we decided to perform EVAR with implantation of an aorto-bisiliac endoprosthesis, using surgical isolation and access of the left common femoral artery and percutaneous access of the right common femoral artery. At the final angiographic control, no endoleak was visible. The following hospital course was completely devoid of complications, without significant post-operative anemia or signs of infections, and the patient was discharged five days after the procedure.

**Figure 1:**
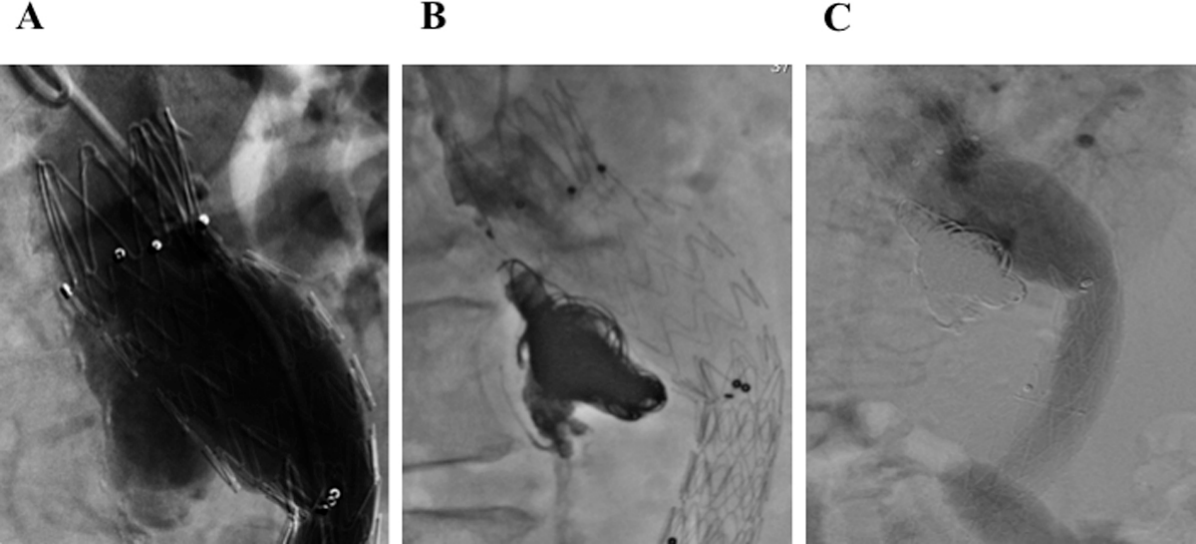
**A. Angiogram following placement of aortic endograft shows a type I endoleak. B. Closure of perigraft endoleak by means of embolization release controlled spirals and liquid embolization Onyx. C. Angiogram showing resolution of Type I endoleak**.

In July 2012, a routine CT control revealed the presence of an apparent type I endoleak. The patient was in a clinically stable state (without any symptoms and no signs of anemia). Aortography confirmed the presence of a posterior peri-graft large leak (Figure 1A). The patient declined the high-risk option of open surgical repair in favor of a final endovascular attempt. He underwent percutaneous closure of the peri-graft endoleak (type I) by means of embolization release controlled spirals and liquid embolization Onyx. Trans-arterial embolization was performed via the radial artery approach. A right radial access was obtained using a micro-puncture kit (Cook Medical), and a 6 Fr sheath (Cordis) was placed in the right radial artery without difficulty. The endoleak channel was engaged with a (SOS Omni) selective catheter. The presence and location of the type I endoleak was confirmed by aortography. The aneurysm sac was treated using embolization release controlled spirals and liquid embolization Onyx (Micro Therapeutics Iac, Irvine, Calif) (Figure 1B). Before injection of Onyx, the microcatheter was flushed with DMSO and Onyx was subsequently injected under fluoroscopy. We used the currently available solutions Onyx-18 (6 % Ethylene vinyl alcohol (EVOH)/94 % DMSO), Onyx-20 (6.5 % EVOH/93.5 % DMSO) or Onyx-34 (8 % EVOH/92 % DMSO). Onyx-18 had the lowest and Onyx-34 the highest viscosity. Post-embolization angiography showed no further endoleak (Figure 1C). At 3-month-follow-up, the patient was free of radiographic evidence of type I endoleak or aneurysm sac espansion.

## Conclusions

Endovascular treatment of aortic aneurysms has progressively improved and has widely replaced the classical surgical procedures [6-8,21-24]. EVAR is widely accepted as a safer and less invasive alternative to open repair. The most common complication of EVAR is endoleak, which occurs when the aortic sac continues to be perfused despite graft fixation. Type I endoleaks are the most frequent and occur at either proximal or distal attachment site or landing zone. These leaks are consequence of a failing apposition of the stent-graft to the cylindrical aortic wall within the landing zone allowing a persistent communication between the pressure-filled aortic lumen and the aneurysm sac or excluded portion of the aortic lumen. The EUROSTAR investigators have also demonstrated that endoleak type I is significantly associated with rupture risk after EVAR [25]. Endovascular technique has significantly decreased mortality rate when compared to open surgery. Endovascular treatment of type I endoleaks requires a complete flow elimination to exclude the aneurysm sac from systemic pressure.

The transradial approach appears to be a safe and effective alternative to the traditional transfemoral approach. The primay benefits of this technique, when compared to femoral artery access, are a decrease in vascular complications, improved patient comfort, earlier ambulation and lower direct costs. Asymptomatic radial artery occlusion, non-occlusive radial artery injury and radial artery spasm are commonly reported complications [26,27]. Symptomatic radial arterial occlusion, pseudoaneurysm and radial artery perforation are much less common. The risk of arterial perforation with the transradial approach is less than 1%, primarily involving the radial artery with the incidence of compartment syndrome at approximately 0.004% [28]. In addition, the risk of significant bleeding requiring transfusion with the transradial approach is extremely rare, occurring in about one patient in a thousand [29].

In this report, we present a case of successful endovascular treatment of type I endoleak by embolization release controlled spirals and liquid embolization Onyx. For trans-arterial embolization, many authors reported the use of microcoils or n-BCA as embolizing materials, but Onyx has shown safety advantages [30]. Onyx is an ethylene vinyl alcohol copolymer dissolved in various concentrations of DMSO and opacified with micronized Tantalum powder for X-ray visualization. When this mixture contacts blood, DMSO diffuses away resulting in solidification of the polymer [31]. Disadvantages of the use of Onyx for endoleak treatment are its high radio-opacity on follow-up CT scans (causing a difficult detection of small persistent endoleaks, comparable to artifacts with coils after endoleak embolization and image subtraction), a relatively long (20 minutes) preparation time, the potential for vasospasm and the high costs [32,33]. Embolization is considered clinically successful when the volume of the aneurysm sac is stable or decreased at follow-up CT scans.

In conclusion, endoleaks remain the primary limitation of endovascular stent-grafting of the abdominal aorta. Type I endoleak is associated with a significantly greater risk of rupture of the aneurysm. Many patients will be able to benefit from endovascular treatment approach to aortic disease through radial arterial access. Larger patient numbers and longer-term follow-up are needed to define procedural efficacy and durability.

## Consent

Written informed consent was obtained from the patient for publication of this case report and any accompanying images. A copy of written consent is available for review by the Editor-in-Chief of this journal.

## Abbreviations

EVAR : Endovascular repair of aortic aneurysms; PAD: Peripheral arterial disease; AAA : Aortic abdominal aneurysms; DMSO : dimethyl sulphoxide; n-BCA : n-butyl 2-cyanoacrylate

## Competing interests

The authors declare that they have no competing interests. No financial support has been received.

## Authors' contributions

GGS, GE and CP acquired the data and wrote/revised the manuscript. FM, FIL, AGB, MDP, AS performed the clinical follow-up of the patient. GE and GGS performed the endovascular treatment. BT and GE approve the final manuscript Furthermore, all authors have been involved in revising the manuscript critically for important intellectual content and read and approved the final manuscript.

## Authors' information

GGS: Resident in Cardiology at University Federico II of Naples.

FM: Resident in Cardiology at University Federico II of Naples.

FIL: Resident in Cardiology at University Federico II of Naples.

RB: Resident in Cardiology at University Federico II of Naples.

AGB: Resident in Cardiology at University Federico II of Naples.

MDP: Resident in Cardiology at University Federico II of Naples.

AS: Resident in Cardiology at University Federico II of Naples.

CP: Assistant Professor of Internal Medicine at University Federico II of Naples.

BA: Associate Professor of Surgery at University "Federico II" of Naples.

DL: Associate Professor of Internal Medicine and Geriatrics at University "Federico II" of Naples.

BT: Full Professor of Cardiology at University Federico II of Naples, Chief Division of Cardiology at University Federico II of Naples.

GE: Associate Professor of Cardiology at University "Federico II" of Naples.

## Declarations

Funding for this article came from personal funds.

This article has been published as part of *BMC Surgery *Volume 13 Supplement 2, 2013: Proceedings from the 26th National Congress of the Italian Society of Geriatric Surgery. The full contents of the supplement are available online at http://www.biomedcentral.com/bmcsurg/supplements/13/S2

## References

[B1] Tendera M Aboyans V Bartelink ML Baumgartner I Clement D Collet JP Cremonesi A De Carlo M Erbel R Fowkes FG ESC Guidelines on the diagnosis and treatment of peripheral artery diseases: Document covering atherosclerotic disease of extracranial carotid and vertebral, mesenteric, renal, upper and lower extremity arteries: the Task Force on the Diagnosis and Treatment of Peripheral Artery Diseases of the European Society of Cardiology (ESC) Eur Heart J 2011 13 2851 2906 21873417 10.1093/eurheartj/ehr211

[B2] Hirsch AT Haskal ZJ Hertzer NR Bakal CW Creager MA Halperin JL Hiratzka LF Murphy WR Olin JW Puschett JB ACC/AHA 2005 guidelines for the management of patients with peripheral arterial disease (lower extremity, renal, mesenteric, and abdominal aortic): executive summary a collaborative report from the American Association for Vascular Surgery/Society for Vascular Surgery, Society for Cardiovascular Angiography and Interventions, Society for Vascular Medicine and Biology, Society of Interventional Radiology, and the ACC/AHA Task Force on Practice Guidelines (Writing Committee to Develop Guidelines for the Management of Patients With Peripheral Arterial Disease) endorsed by the American Association of Cardiovascular and Pulmonary Rehabilitation; National Heart, Lung, and Blood Institute; Society for Vascular Nursing; TransAtlantic Inter-Society Consensus; and Vascular Disease Foundation J Am Coll Cardiol 2006 13 1239 1312 16545667 10.1016/j.jacc.2005.10.009

[B3] Schiano V Sirico G Giugliano G Laurenzano E Brevetti L Perrino C Brevetti G Esposito G Femoral plaque echogenicity and cardiovascular risk in claudicants JACC Cardiovasc Imaging 2012 13 348 357 22498323 10.1016/j.jcmg.2012.01.011

[B4] Giugliano G Sannino A Brevetti L Perrino C Schiattarella GG Franzone A Serino F Ferrone M Scudiero F Carbone A Ankle/brachial index to everyone BMC Surg 2012 13 Suppl 1 S18 23173985 10.1186/1471-2482-12-S1-S18 PMC3499282

[B5] Gargiulo G Giugliano G Brevetti L Sannino A Schiattarella GG Serino F Carbone A Scudiero F Ferrone M Corrado R Use of statins in lower extremity artery disease: a review BMC Surg 2012 13 Suppl 1 S15 23173874 10.1186/1471-2482-12-S1-S15 PMC3499199

[B6] Giugliano G Di Serafino L Perrino C Schiano V Laurenzano E Cassese S De Laurentis M Schiattarella GG Brevetti L Sannino A Effects of successful percutaneous lower extremity revascularization on cardiovascular outcome in patients with peripheral arterial disease Int J Cardiol 2012 10.1016/j.ijcard.2012.06.055 22790191

[B7] Ilardi F Magliulo F Gargiulo G Schiattarella GG Carotenuto G Serino F Ferrone M Visco E Scudiero F Carbone A Endovascular treatment of carotid artery stenosis: evidences from randomized controlled trials and actual indications Monaldi Arch Chest Dis 2011 13 183 191 22567734 10.4081/monaldi.2011.175

[B8] Perrino C Scudiero L Petretta MP Schiattarella GG De Laurentis M Ilardi F Magliulo F Carotenuto G Esposito G Total occlusion of the abdominal aorta in a patient with renal failure and refractory hypertension: a case report Monaldi Arch Chest Dis 2011 13 43 46 21751737 10.4081/monaldi.2011.205

[B9] Giugliano G Laurenzano E Rengo C De Rosa G Brevetti L Sannino A Perrino C Chiariotti L Schiattarella GG Serino F Abdominal aortic aneurysm in patients affected by intermittent claudication: prevalence and clinical predictors BMC Surg 2012 13 Suppl 1 S17 23173942 10.1186/1471-2482-12-S1-S17 PMC3499243

[B10] Ebaugh JL Garcia ND Matsumura JS Screening and surveillance for abdominal aortic aneurysms: who needs it and when Semin Vasc Surg 2001 13 193 199 11561280 10.1053/svas.2001.25491

[B11] Wilmink TB Quick CR Day NE The association between cigarette smoking and abdominal aortic aneurysms J Vasc Surg 1999 13 1099 1105 10587395 10.1016/s0741-5214(99)70049-2

[B12] Lindholt JS Juul S Fasting H Henneberg EW Screening for abdominal aortic aneurysms: single centre randomised controlled trial BMJ 2005 13 750 15757960 10.1136/bmj.38369.620162.82 PMC555873

[B13] Brown PM Pattenden R Gutelius JR The selective management of small abdominal aortic aneurysms: the Kingston study J Vasc Surg 1992 13 21 25 discussion 25-27 1728677 10.1067/mva.1992.33840

[B14] Huber TS Wang JG Derrow AE Dame DA Ozaki CK Zelenock GB Flynn TC Seeger JM Experience in the United States with intact abdominal aortic aneurysm repair J Vasc Surg 2001 13 304 310 discussion 310-301 11174782 10.1067/mva.2001.112703

[B15] Heller JA Weinberg A Arons R Krishnasastry KV Lyon RT Deitch JS Schulick AH Bush HL Kent KC Two decades of abdominal aortic aneurysm repair: have we made any progress? J Vasc Surg 2000 13 1091 1100 11107080 10.1067/mva.2000.111691

[B16] Parodi JC Palmaz JC Barone HD Transfemoral intraluminal graft implantation for abdominal aortic aneurysms Ann Vasc Surg 1991 13 491 499 1837729 10.1007/BF02015271

[B17] Veith FJ Baum RA Ohki T Amor M Adiseshiah M Blankensteijn JD Buth J Chuter TA Fairman RM Gilling-Smith G Nature and significance of endoleaks and endotension: summary of opinions expressed at an international conference J Vasc Surg 2002 13 1029 1035 12021724 10.1067/mva.2002.123095

[B18] Gallo P Latronico MV Gallo P Grimaldi S Borgia F Todaro M Jones P Gallinari P De Francesco R Ciliberto G Inhibition of class I histone deacetylase with an apicidin derivative prevents cardiac hypertrophy and failure Cardiovasc Res 2008 13 416 424 18697792 10.1093/cvr/cvn215

[B19] Riccio G Esposito G Leoncini E Contu R Condorelli G Chiariello M Laccetti P Hrelia S D'Alessio G De Lorenzo C Cardiotoxic effects, or lack thereof, of anti-ErbB2 immunoagents FASEB J 2009 13 3171 3178 19417081 10.1096/fj.09-131383

[B20] Chun JY Morgan R Transcatheter embolisation of type 1 endoleaks after endovascular aortic aneurysm repair with Onyx: when no other treatment option is feasible Eur J Vasc Endovasc Surg 2013 13 141 144 23276679 10.1016/j.ejvs.2012.11.010

[B21] Esposito G Franzone A Cassese S Schiattarella GG Capretti G Pironti G Di Serafino L Perrino C Piscione F Chiariello M Endovascular repair for isolated iliac artery aneurysms: case report and review of the current literature J Cardiovasc Med (Hagerstown) 2009 13 861 865 19543108 10.2459/JCM.0b013e32832e1904

[B22] Esposito G Di Serafino L Gargiulo G Sannino A Schiattarella GG Franzone A Perrino C Chiariello M Rotational atherectomy for the treatment of isolated femoral artery traumatic lesion: A case report Monaldi Archives for Chest Disease - Cardiac Series 2009

[B23] Amato B Iuliano GP Markabauoi AK Piscitelli V Masone S Compagna R Esposito G Piscione F Endovascular procedures in critical leg ischemia of elderly patients Acta Biomed 2005 13 Suppl 1 11 15 16450500

[B24] Giugliano G Perrino C Schiano V Brevetti L Sannino A Schiattarella GG Gargiulo G Serino F Ferrone M Scudiero F Endovascular treatment of lower extremity arteries is associated with an improved outcome in diabetic patients affected by intermittent claudication BMC Surg 2012 13 Suppl 1 S19 23174008 10.1186/1471-2482-12-S1-S19 PMC3499211

[B25] Buth J Harris PL van Marrewijk C Fransen G The significance and management of different types of endoleaks Semin Vasc Surg 2003 13 95 102 12920679 10.1016/s0895-7967(03)00007-3

[B26] Schueler A Black SR Shay N Management of Transradial Access for Coronary Angiography J Cardiovasc Nurs 2012 10.1097/JCN.0b013e3182648351 22990233

[B27] Shroff A Siddiqui S Burg A Singla I Identification and management of complications of transradial procedures Curr Cardiol Rep 2013 13 350 23420446 10.1007/s11886-013-0350-x

[B28] Masuda N Matsukage T Ikari Y Successful transradial intervention for two lesions with dual anomalous origins of coronary arteries J Invasive Cardiol 2011 13 E117 120 21562358

[B29] Kanei Y Kwan T Nakra NC Liou M Huang Y Vales LL Fox JT Chen JP Saito S Transradial cardiac catheterization: a review of access site complications Catheter Cardiovasc Interv 2011 13 840 846 21567879 10.1002/ccd.22978

[B30] Grisafi JL Boiteau G Detschelt E Potts J Kiproff P Muluk SC Endoluminal treatment of type IA endoleak with Onyx J Vasc Surg 2010 13 1346 1349 20655689 10.1016/j.jvs.2010.06.021

[B31] Peynircioglu B Turkbey B Ozkan M Cil BE Use of glue and microcoils for transarterial catheter embolization of a type 1 endoleak Diagn Interv Radiol 2008 13 111 115 18553288

[B32] Maldonado TS Rosen RJ Rockman CB Adelman MA Bajakian D Jacobowitz GR Riles TS Lamparello PJ Initial successful management of type I endoleak after endovascular aortic aneurysm repair with n-butyl cyanoacrylate adhesive J Vasc Surg 2003 13 664 670 14560210 10.1016/s0741-5214(03)00729-8

[B33] Weber W Kis B Siekmann R Kuehne D Endovascular treatment of intracranial arteriovenous malformations with onyx: technical aspects AJNR Am J Neuroradiol 2007 13 371 377 17297015 PMC7977391

